# A Primate Specific Extra Domain in the Molecular Chaperone Hsp90

**DOI:** 10.1371/journal.pone.0071856

**Published:** 2013-08-09

**Authors:** Vishwadeepak Tripathi, Wolfgang M. J. Obermann

**Affiliations:** Ruhr-University Bochum, Institute for Physiology, Bochum, Germany; St. Georges University of London, United Kingdom

## Abstract

Hsp90 (heat shock protein 90) is an essential molecular chaperone that mediates folding and quality control of client proteins. Many of them such as protein kinases, steroid receptors and transcription factors are involved in cellular signaling processes. Hsp90 undergoes an ATP hydrolysis dependent conformational cycle to assist folding of the client protein. The canonical Hsp90 shows a typical composition of three distinct domains and interacts with individual cochaperone partners such as Hop, Cdc37 and Aha1 (activator of Hsp90 ATPase) that regulate the reaction cycle of the molecular chaperone. A bioinformatic survey identified an additional domain of 122 amino acids in front of the canonical Hsp90 sequence. This extra domain (E domain) is specific to the Catarrhini or drooping nose monkeys, a subdivision of the higher primates that includes man, the great apes and the old world monkeys but is absent from all other species. Our biochemical analysis reveals that Hsp103 associates with cochaperone proteins such as Hop, Cdc37 and Aha1 similar to Hsp90. However, the extra domain reduces the ATP hydrolysis rate to about half when compared to Hsp90 thereby acting as a negative regulator of the molecular chaperonés intrinsic ATPase activity.

## Introduction

Molecular chaperone systems such as Hsp70, Hsp90 and TRiC oversee the folding and quality control of many proteins and assist their folding or triage them for clearance mediated by the proteolytic machinery to maintain cellular proteostasis [1]. Heat shock protein 90 (Hsp90) is considered a specialized folding tool to control client proteins that are mainly involved in signaling processes such as protein kinases, steroid hormone receptors and transcription factors [2] and regulate progression through the cell cycle. Since mutations in many client proteins are oncogenic and associated with neoplastic transformation of the cell, Hsp90 has gained importance as a target for drugs that inhibit the molecular chaperone and thereby prevent client protein activation [3], [4].

Hsp90 undergoes a conformational cycle driven by the hydrolysis of ATP during which the client protein attains its native conformation [5]. In doing so, Hsp90 operates in an orchestrated fashion together with cochaperone partners that regulate its activity in several ways. The molecular chaperone consists of three building blocks, an N-terminal ATP binding domain (Hsp90N), a middle domain (Hsp90M) and a C-terminal domain (Hsp90C), that mediates dimerization of the molecule [6]. Amongst the various cochaperones are several tetratricopeptide (TPR) repeat domain proteins that bind at the chaperonés C-terminal domain, such as Hop that links Hsp90 to the Hsp70 chaperone system [5]. Other cochaperones such as Cdc37 are involved in the regulation of protein kinases and use unique motifs for binding to N-terminal part of Hsp90 [5]. Yet another cochaperone, Aha1 (activator of Hsp90 ATPase), associates with the middle domain of Hsp90 and stimulates its ATPase activity [7], [8]. Recent studies point to the view that Aha1 may be involved in recognition of folding defective proteins such as mutant CFTRΔF508 that causes the respiration disease Cystic Fibrosis [9], [10].

A recent inspection of the database revealed a gene product that contained 122 additional amino acids (aa) fused to the N-terminus of the canonical human Hsp90α sequence. We find that this enlarged protein, named Hsp103, is present only in higher old world primates such as rhesus macaque, chimpanzee and man but absent from other mammals and any other organisms. On the level of purified proteins Hsp103 interacts with cochaperone proteins such as Hop, Cdc37 and Aha1, similar to Hsp90α. In contrast, Hsp103 shows only roughly half of the ATPase activity when compared to Hsp90α.

## Materials and Methods

### Analytical and quantitative PCR (qPCR)

Hsp103 and Hsp90α sequences were amplified from a human multiple tissue cDNA panel (Clontech) using Titanium Taq polymerase. Primer sequences were: P1 5′-ATG CCC CCG TGT TCG GGC GGG GAC GGC TCC ACC CCT CCT-3′, P2 5′-AGA GTC TAA TTT ACT GGG ATC TGT CAA GCT TTC ATA CCG-3′, P3 5′-AGC AGG GCA CCT GTT AAC TGG TAC CAA GAA AAG GCC CAA-3′, P4 5′-CAG TAG CCT AAG CAA TAT AAA TGG CTG CAG ATC CTT GTA-3′, P5 5′-ATC TGG ACC AGA AAT CCC GAC GAT ATT ACT AAT GAG GAG-3′ and P6 5′-CTT GTA GTT CTC TTT ATC TTC CGC CAG TTC AGT AAA GAG-3′; GAPDH (glyceraldehyde phosphate dehydrogenase) specific primers 5′-TGA AGG TCG GAG TCA ACG GAT TTG GT-3′ and 5′-CAT GTG GGC CAT GAG GTC CAC CAC-3′ served as a control.

**Figure 1:**
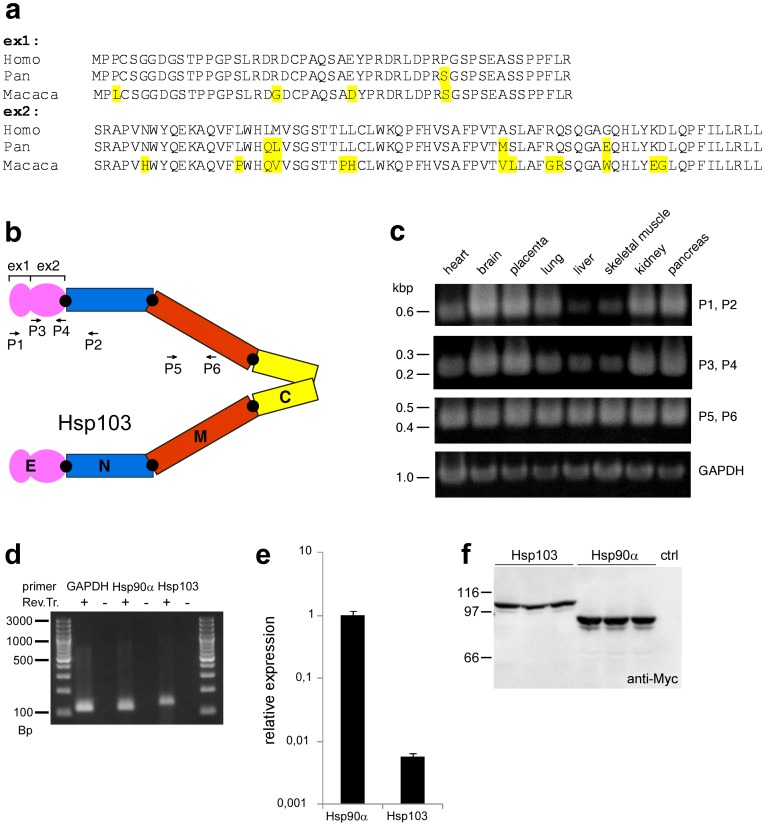
The molecular chaperone Hsp103 contains a fourth domain of 122 aa, termed E domain. (a) Alignment of E domain sequences from man (Homo sapiens), chimpanzee (Pan troglodytes) and rhesus macaque (Macaca mulatta). Residues that differ between the species are marked in grey. (b) Four domain structure of Hsp103. The additional 122 aa encoded by the two exons ex1 and ex2 form the E domain. Positions of Primers (P1–P6) used for PCR analysis are indicated. (c) Amplification of Hsp103 from a human multiple tissue cDNA panel. Primers P1, P2 amplify a contiguous sequence covering the E domain and parts of the N domain. P3, P4 amplify ex2 of Hsp103 and P5, P6 amplify a sequence in the M domain present in Hsp90α and Hsp103. GAPDH is shown as a control. (d) The three primer probes used for qPCR produce single bands for GAPDH, Hsp90α and Hsp103 indicating their specificity. No signals were visible on samples without Reverse Transcriptase (Rev. Tr.), confirming the absence of contaminating genomic material. (e) Expression of endogenous Hsp103 mRNA is below 1% compared to Hsp90α on a logarithmic scale as revealed by qPCR. (f) Expression of Hsp103 and Hsp90α with N-terminal Myc-tag from the pCMV plasmid in HEK293 cells. Transfections were done in triplicate; control cells received empty vector. Proteins were visualized by Western blotting with the Myc specific antibody 9E10.

To analyze Hsp103 expression levels by qPCR the specific oligo nucleotide probes 5′-CTT CGG GAC AGG GAC TGT CCC GCC-3′ (sense) directed against exon 1 and 5′-TTG GGC CTT TTC TTG GTA CCA GTT-3′ (antisense) directed against exon 2 of the E domain were used. To amplify Hsp90α transcripts and the internal standard GAPDH, respectively, we used validated QuantiTect primer assays Hs_HSP90AA1_3_SG and Hs-GAPDH_2_SG (Qiagen). The Hs_HSP90AA1_3_SG probe detects Hsp90α and the longer transcript Hsp103. However, since Hsp103 expression is below 1% of Hsp90α as shown in Fig. 1e, quantification using this probe gives an accurate measure of Hsp90α levels. For total RNA purification using the RNeasy kit (Qiagen), confluent HEK293 cells from one well of a 24 well plate were lysed in 225 µl buffer. 4 µl of this lysate were used for first strand cDNA synthesis in a total of 20 µl using the QuantiTect reverse transcription kit (Qiagen). Finally, 0.5 µl thereof were used in the qPCR experiments. qPCR was carried out on a MJ Research Opticon 2 real-time cycler using the SYBR Green Advantage qPCR premix (Clontech). We corrected for the efficiency of the E domain and Hsp90α primer probes on serial dilutions of human Hsp103 cDNA and used GAPDH as an internal standard between samples.

### Bioinformatics and database mining

We performed BLAST searches against all mammalian genomes (www.ncbi.nlm.nih.gov/genomes/leuks.cgi?p3=12:Mammals&taxgroup=11:|12:Mammals) using the amino acid (UniProtKB P07900-2) and nucleotide sequences (NCBI NM_001017963) encoding the E domain of Hsp103 and regular Hsp90α as a positive control.

### Construction of expression plasmids

Prokaryotic expression plasmids containing human Hsp90α, Hop, Aha1 and Cdc37 have been described elsewhere [7], [11]–[13]. N-terminal sequences encoding the additional E domain were amplified from a human brain cDNA library (Clontech) and the expression plasmid for human Hsp103 obtained by fusion to the Hsp90α sequence in the pProExHTa vector using the NcoI site of the vectoŕs multiple cloning site and the internal BglII site of the Hsp90α sequence. All constructs were verified by DNA sequencing.

### Cell culture experiments

For expression in HEK293 cells with an N-terminally attached Myc-tag, human Hsp90α and Hsp103 cDNAs were subcloned from pProExHTa vectors decribed above into the pCMV vector and transfected using Lipofectamine 2000 (Life Technologies). Protein expression was detected with the monoclonal antibody 9E10 specific for the Myc epitope.

### Protein purification

E. coli BL21(DE3)pLsyS cells were transformed with chaperone and cochaperone constructs in the pProExHTa vector and protein expression induced with 0.2 mM IPTG at 18 or 25°C for 5 – 16 h. Proteins were purified on Nickel-nitriloacetate chelate affinity resin and ResourceQ ion exchange chromatography on an ÄktaPurifier system (GE Healthcare), as reported previously [12], [14].

### Protein interaction assays

Chaperones alone or in combination with the indicated cochaperone proteins at a concentration of 2.5 µM, as determined by the Bradford reagent, were incubated for 10 min at 30°C following 10 min on ice in 40 mM Hepes-KOH buffer pH 7.4, 50 mM KCl and 2 mM Mg_2_Cl. Protein samples were separated on a Superdex 200 HR 10/30 column equilibrated in the same buffer using an ÄktaPurifier system. Fractions (500 µl) were collected and analyzed by SDS-PAGE on 10% gels. Antibodies ab56721 (Abcam) specific for Aha1 and sc-13129 (Santa Cruz Biotechnology) specific for Cdc37 were used for Western blotting.

### Measurement of ATPase activity

Hsp90 ATPase activity was measured essentially as described by a coupled enzymatic system [15]. Briefly, ADP produced by the molecular chaperone is re-phosphorylated to ATP by pyruvate kinase (PK) that converts phosphoenolpyruvate to pyruvate. Pyruvate is reduced to lactate by lactate dehydrogenase (LDH) that oxidizes NADH to NAD^+^ and this reaction can be monitored by the decrease in absorption at 340 nm. Since PK and LDH catalyzed reactions are fast compared to Hsp90 dependent ATP hydrolysis, the ATPase rate is directly coupled to NADH consumption and to the decrease of absorption at 340 nm.

ATPase experiments were performed in 40 mM Hepes-KOH buffer pH 7.4, 2 mM Mg_2_Cl and contained 2.5 µM of Hsp90α or Hsp103, 0.1 µg/µl of PK and LDH (Roche), 1 mM ATP, 2 mM phosphoenol pyruvate, 0.1 mM NADH and 1% DMSO. Parallel assays received 100 µM Radicicol in 1% DMSO to inhibit the Hsp90α and Hsp103 ATPase and measure background activity. Thus, subtraction of two corresponding experiments in the presence or absence of Radicicol allows determining the specific ATP hydrolysis rate of Hsp90α and Hsp103.

## Results and Discussion

### An additional domain fused to Hsp90α

It is well established that the molecular chaperone Hsp90 with its typical three domain composition is conserved from bacteria to man with an essential role in all eukaryotic cells. Surprisingly, a recent inspection of the database revealed an isoform of human Hsp90α with an additional sequence of 122 amino acids (aa) at the N-terminus (amino acid sequence: UniProtKB P07900-2; cDNA sequence: NCBI NM_001017963) (Fig. 1a, b). The extra 122 aa are encoded by 2 exons in front of the canonical human Hsp90α DNA and separated by 2 large introns of 37 and 15 kBp [16]. Alternative splicing generates the gene products Hsp90AA1-1 ( =  Hsp90α, 732 aa) and the larger Hsp90AA1-2 (852 aa) [16]. To facilitate nomenclature, we address the additional 122 aa sequence as E domain (extra domain) and the longer protein consisting of 854 aa as Hsp103, referring to the ∼13 kDa in size added to Hsp90α, throughout this text. A similar N-terminal extension is not present in the highly homologous Hsp90β protein.

We have analyzed the expression of Hsp103 using a human multiple tissue cDNA panel (Clontech) by PCR (polymerase chain reaction) analysis using Titanium Taq polymerase (Clontech) (Fig. 1c). Interestingly, we detected considerable signals for Hsp103 in brain, placenta, kidney and pancreas while signals in heart, lung, liver and skeletal muscle were faint with both primer pairs P1, P2 and P3, P4. In contrast, the primer pair P5, P6 that amplifies Hsp90α sequences in the M domain generates signals in all 8 tissues, similar to the glycerinaldehyde phosphate dehydrogenase (GAPDH) control (Fig. 1c). To obtain a quantitative measure of Hsp103 mRNA expression in comparison to Hsp90α, we employed quantitative real-time PCR (qPCR). It turns out, that endogenous Hsp103 mRNA expression in HEK293 cells is below 1% compared to Hsp90α (Fig. 1d, e), suggesting that endogenous Hsp103 mRNA expression is tightly regulated and may play a specific role beyond that of Hsp90α. Remarkably, Hsp90β lacks the additional N-terminal sequence and therefore one may speculate that the presence of the E domain is related to the fact that Hsp90α is stress inducible and Hsp90β not.

To rule out that the additional sequence of the Hsp103 cDNA is a non-translated aberration, we tested protein expression (Fig. 1f). Therefore, we cloned Hsp103 and Hsp90α in the pCMV vector and transfected the construct into HEK293 cells. Blotting with a Myc tag specific antibody revealed that Hsp103 migrates at the expected size, slower than Hsp90α, confirming that the additional domain is expressed in this human cell line (Fig. 1f). Hsp103 should be theoretically detectable with an Hsp90α specific antibody and we have used human HEK293 cells and rhesus macaque brain and liver as a source to test this. However, Hsp103 and Hsp90α are too similar in size so that both proteins can hardly be separated and therefore the overwhelming amount of Hsp90α generated such a strong western blot signal that made detection of a hypothetical, but low abundant, slightly slower migrating band impossible. Furthermore, efforts to generate peptide specific antibodies against E domain sequences failed. However, our evidence for Hsp103 is based on the following three independent indications: the presence of mRNA specific for Hsp103 in 8 different human tissues; expression of Hsp103 similar to Hsp90α in cells from a plasmid; and the presence of sequences encoding the additional E domain of Hsp103 in man and four additional higher primates (see below).

### Hsp103 is present in higher primates only

Hsp90α is a universal molecular chaperone present in all eukaryotes and we were wondering whether this is also true for Hsp103. BLAST analysis revealed that sequences coding for the Hsp103 specific E domain are absent from all unicellular organisms, plants, insects, worms, fish, amphibians, reptiles, birds and mammals including opossum, platypus, pig, cow, horse, dog, cat, sheep, mouse, rat and rabbit.

**Figure 2:**
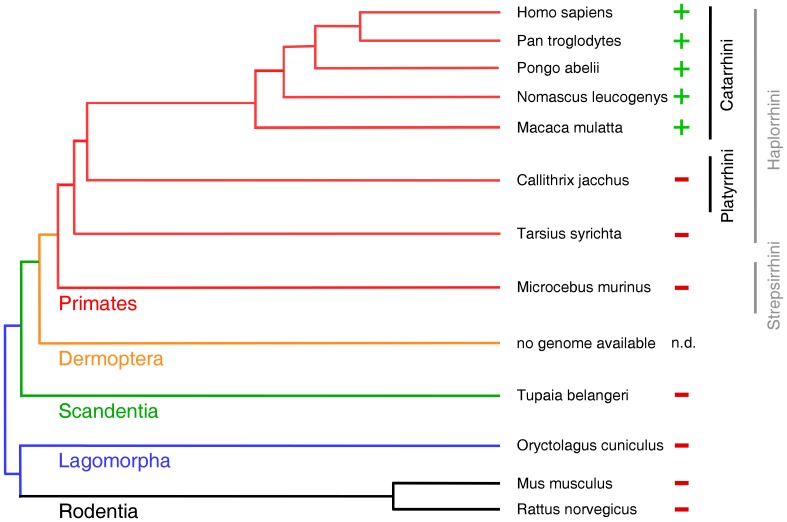
The molecular chaperone Hsp103 is specific to higher primates. The supraorder Euarchontoglires comprises the orders Primates, Dermoptera, Scandentia, Lagomorpha and Rodentia. This illustration shows the relationship of the species which we analyzed for Hsp103 E domain sequences. The figure is a simplified re-drawing from the comprehensive phylogenetic trees generated by Janecka et al. [22] and Perelman et al. [23], limited to the 12 species shown. The presence or absence the extra E domain sequence in the respective species is indicated by + or –. It turned out that the extra domain of Hsp103 is only present in the genome of Catarrhini, a primate suborder that contains the old world monkeys, great apes and man. Genomic data for the order Dermoptera were not available (n.d.). E domain specific sequences were detected by BLAST searches as described in Materials and Methods for Nomascus leucogenys under NCBI XP_003276214; for Pan troglodytes under NCBI NW_003457860.1 (regions from base 6775968 to 6776180 and from 6814113 to 6814267, matching exon2 and exon1); for Pongo abelii under NCBI NW_002887407.1 (regions from base 9860 to 10072 and from 50476 to 50631) and for Macaca mulatta under NCBI NW_001121227.1 (regions from base 1585406 to 1585618 and from 1619418 to 1619580).

A survey of the superorder Euarchontoglires (Supraprimates) revealed that Hsp103 with the extra E domain is present only in primates such as man (Homo sapiens), chimpanzee (Pan troglodytes), Northern white-cheeked gibbon (Nomascus leucogenys), orangutan (Pongo abelii) and rhesus macaque (Macaca mulatta) but absent from other primates such as marmoset (Callithrix jacchus), Philippine tarsier (Tarsius syrichta) and gray mouse lemur (Microcebus murinus) (Fig. 2). Furthermore, we could not detect Hsp103 in species representing the orders Rodentia (mouse, Mus musculus; rat, Rattus norvegicus), Lagomorpha (rabbit, Oryctolagus cuninculus) and Scandentia (Northern tree shrew, Tupaia belangeri). Data for the order Dermoptera are currently not available. Thus, our analysis suggests that Hsp103 is specific to the Catarrhini, also called drooping nose monkeys, a subdivision of the higher primates that includes the old world monkeys, the great apes and man. Given that Hsp103 levels are far beyond that of Hsp90α (Fig. 1e), the E domain may serve a special role that may have emerged during primate evolution.

### Hsp103 binds to cochaperone proteins similar to Hsp90α

**Figure 3:**
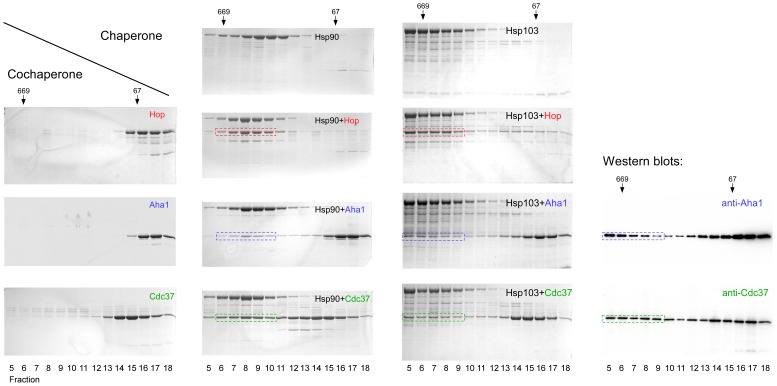
Interaction of Hsp103 with cochaperone proteins. Purified Hsp90α, Hsp103, Hop, Aha 1 and Cdc37 were mixed as indicated and fractionated by gel filtration chromatography on a Superdex 200 column. Fractions were analyzed by SDS-PAGE. Marker proteins are shown on top (thyroglobulin, 669 kDa; BSA, 67 kDa). The shift of the cochaperones Hop, Aha1 and Cdc37 indicating complex formation with Hsp90α and Hsp103 is marked by red, blue or green dashed lines, respectively. For the Hsp103+Aha1 and Hsp103+Cdc37 interaction runs the cochaperones were blotted with specific antibodies to prove the identity of the shifted Aha1 and Cdc37 bands that indicate complex formation with Hsp103.

The additional E domain may regulate chaperone features like binding of cochaperone proteins or the ATP hydrolysis rate of Hsp90. Therefore, we purified recombinant Hsp103 and Hsp90α to analyze their interaction with the cochaperone proteins Hop, Aha1 and Cdc37. Since Hop binds to Hsp90C [17], this interaction should be unaffected by the additional domain of Hsp103. On the other hand, Cdc37 that binds to Hsp90N [18] and Aha1 that binds to Hsp90M and makes contacts to Hsp90N [7], [19], [20] might sense the presence of the E domain which could then impact its association with the molecular chaperone. We used gel filtration chromatography to analyze complex formation between the chaperones Hsp103 and Hsp90α, and the cochaperones Hop, Aha1 and Cdc37 (Fig. 3). A shift in the elution profile of the cochaperone towards higher molecular mass compared to the single protein indicates the formation of chaperone-cochaperone complexes. It turned out that Hsp90α binds to the Hop, Aha1 and Cdc37 cochaperone proteins as expected (Fig. 3). While the interactions with Hop and Cdc37 are quite robust, a minor amount of Aha1 shifts with Hsp90α, indicating that this complex is more transient. These observations are in agreement with previous data where yeast Hsp90 and Aha1 were analyzed by the same technique [7], [12]. When Hsp103 instead of Hsp90α was mixed with the cochaperone proteins, a similar result was obtained (Fig. 3). Hop`s and Cdc37`s binding to Hsp103 is robust while Aha1 interacts with Hsp103 like with Hsp90α. These results suggest that the E domain does not alter the association with Hop, Cdc37 and Aha1 but may impact the molecular chaperone in yet another way.

### The E domain decreases the intrinsic ATP hydrolysis rate of Hsp103 compared to Hsp90α

**Figure 4:**
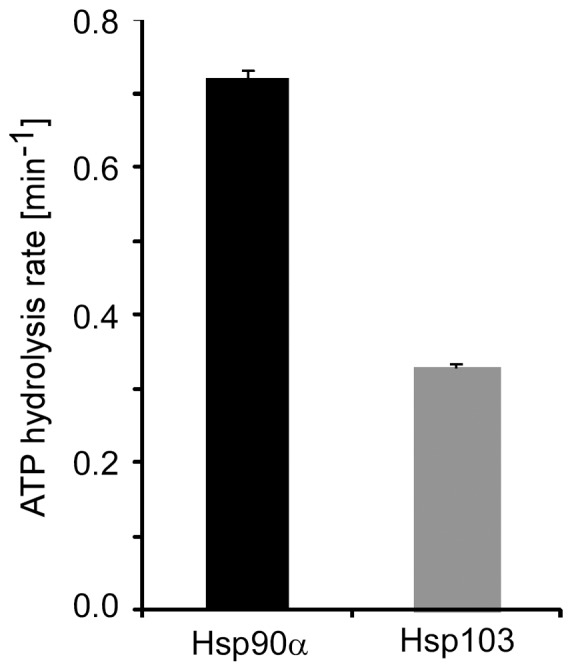
The E domain acts as a negative regulator of Hsp103′s intrinsic ATPase activity. The ATP hydrolysis rates of human Hsp90α and Hsp103 with the extra domain was measured. Three independent experiments were averaged and the standard deviation depicted by error bars.

ATP hydrolysis is a hallmark of Hsp90 and it is well established that this activity is required for the in vivo function of the molecular chaperone [15], [21]. Therefore, our aim was measuring to which extent the ATPase activity can be modulated by the E domain. We determined the intrinsic ATP hydrolysis rate of Hsp90α and Hsp103 as described in Materials and Methods. Remarkably, Hsp103 showed only roughly half of the specific ATPase activity compared to Hsp90α (Fig. 4) supporting the conclusion that the additional domain acts as a negative regulating element of Hsp103′s intrinsic hydrolysis activity. Thus, Hsp103′s ATPase driven conformational cycling may be adjusted to assist client proteins that fold slower than others that are dependent on the canonical Hsp90α.
